# Recent advances and future perspectives in the therapeutics of prostate cancer

**DOI:** 10.1186/s40164-023-00444-9

**Published:** 2023-09-22

**Authors:** Ganji Lakshmi Varaprasad, Vivek Kumar Gupta, Kiran Prasad, Eunsu Kim, Mandava Bhuvan Tej, Pratik Mohanty, Henu Kumar Verma, Ganji Seeta Rama Raju, LVKS Bhaskar, Yun Suk Huh

**Affiliations:** 1 Department of Biological Sciences and Bioengineering, Biohybrid Systems Research Center (BSRC), Inha University, Incheon, 22212 Republic of Korea; 2 Department of Zoology, Guru Ghasidas Vishwavidyalaya, Bilaspur, India; 3 Department of Health Care Informatics, Sacred Heart University, 5151 Park Avenue, Fair Fields, CT 06825 USA; 4 Department of Immunopathology, Institute of Lungs Health and Immunity, Helmholtz Zentrum, 85764 Neuherberg, Munich Germany; 5 Department of Energy and Materials Engineering, Dongguk University-Seoul, Seoul, 04620 Republic of Korea

**Keywords:** Prostate cancer, Risk factors, Diagnosis, Treatment, Quality of life

## Abstract

Prostate cancer (PC) is one of the most common cancers in males and the fifth leading reason of death. Age, ethnicity, family history, and genetic defects are major factors that determine the aggressiveness and lethality of PC. The African population is at the highest risk of developing high-grade PC. It can be challenging to distinguish between low-risk and high-risk patients due to the slow progression of PC. Prostate-specific antigen (PSA) is a revolutionary discovery for the identification of PC. However, it has led to an increase in over diagnosis and over treatment of PC in the past few decades. Even if modifications are made to the standard PSA testing, the specificity has not been found to be significant. Our understanding of PC genetics and proteomics has improved due to advances in different fields. New serum, urine, and tissue biomarkers, such as PC antigen 3 (PCA3), have led to various new diagnostic tests, such as the prostate health index, 4K score, and PCA3. These tests significantly reduce the number of unnecessary and repeat biopsies performed. Chemotherapy, radiotherapy, and prostatectomy are standard treatment options. However, newer novel hormone therapy drugs with a better response have been identified. Androgen deprivation and hormonal therapy are evolving as new and better options for managing hormone-sensitive and castration-resistant PC. This review aimed to highlight and discuss epidemiology, various risk factors, and developments in PC diagnosis and treatment regimens.

## Introduction

Cancer is characterized by uncontrolled proliferation, in which the cell loses its regulated division, differentiation, and apoptosis. Cancer is a global burden and a leading cause of mortality and decreasing life expectancy worldwide [1]. According to the International Agency for Research on Cancer, nearly 19.3 million new cancer cases and approximately 10 million cancer-related deaths are expected by 2020. The five most commonly diagnosed cancers are female breast cancer (11.7%), lung cancer (11.4%), colorectal cancer (10%), prostate cancer (PC; 7.3%), and stomach cancer (5.6%) [2].

PC is a non-cutaneous cancer most commonly seen in males after 50 years of age, affecting nearly 1.6 million individuals with more than 3,00,000 deaths worldwide [3]. Reports showed that it is the second most commonly identified cancer in males and the fifth major cause of cancer-related death [4]. In India, PC was one of the most frequently detected cancers in 2020, with 41,532 new cases, accounting for 5.7% of the total cancer cases in men, with one in 125 men at risk of being diagnosed with PC [5]. The higher incidence rate of PC in the last few decades has been mainly due to increased prostate-specific antigen (PSA) screening, leading to a decrease in mortality among diagnosed cases [6–10]. Early diagnosis and improvements in treatment strategies are major factors in the decline in mortality rates [11]. The prostate is a walnut-sized gland present in the male pelvis, which secretes seminal fluid and releases an alkaline solution that helps sperm to survive in the acidic environment of the vagina and helps in nourishing and transporting sperm [12]. Some of the different types of PC found in men include adenocarcinomas, squamous cell carcinomas, transitional cell carcinomas, neuroendocrine tumors, and prostate sarcomas. The most frequent type of PC is adenocarcinoma (90–95%). Age is the most common risk factor for the development of PC, as the incidence rate increases in those over 50 years old. Other associated risk factors includes race and ethnicity, diet, obesity, family history, and smoking [14].

The clinical symptoms of PC depend on the cancer stage, that is, whether the PC is early or advanced. The most commonly observed symptoms include urinary tract signs and symptoms such as painful and poor urinary stream, frequent urination, erectile dysfunction, painful ejaculation, and hematuria [15]. Metastasis of PC to the vertebrae can lead to Pott’s disease, with chronic back and hip pain reported in patients. Furthermore, urinary incontinence has been observed after radical prostatectomy in the early stages of PC [16–18]. For the early detection of PC, PSA biomarker screening has been recommended for the men aged 55–69 years, with a digital rectal examination (DRE) performed in patients with a high PSA level [19]. A systemic prostate biopsy can then be conducted for the final analysis of adenocarcinoma using transrectal ultrasound (TRUS) or transperineal biopsy or multiparametric magnetic resonance imaging (mpMRI) or targeted MRI-ultrasound fusion biopsy [20–22]. The Gleason grading system is used to grade the tumor, which helps the patient choose the correct therapeutic options [23]. A new diagnostic approach uses mp-MRI before the biopsy, which helps detect PC in biopsy-naive patients [24]. Non-invasive diagnostic tools, such as liquid biopsy, can also be used for detection of PC [25]. These diagnostic techniques are mainly adopted by physicians for tumor detection.

**Fig. 1:**
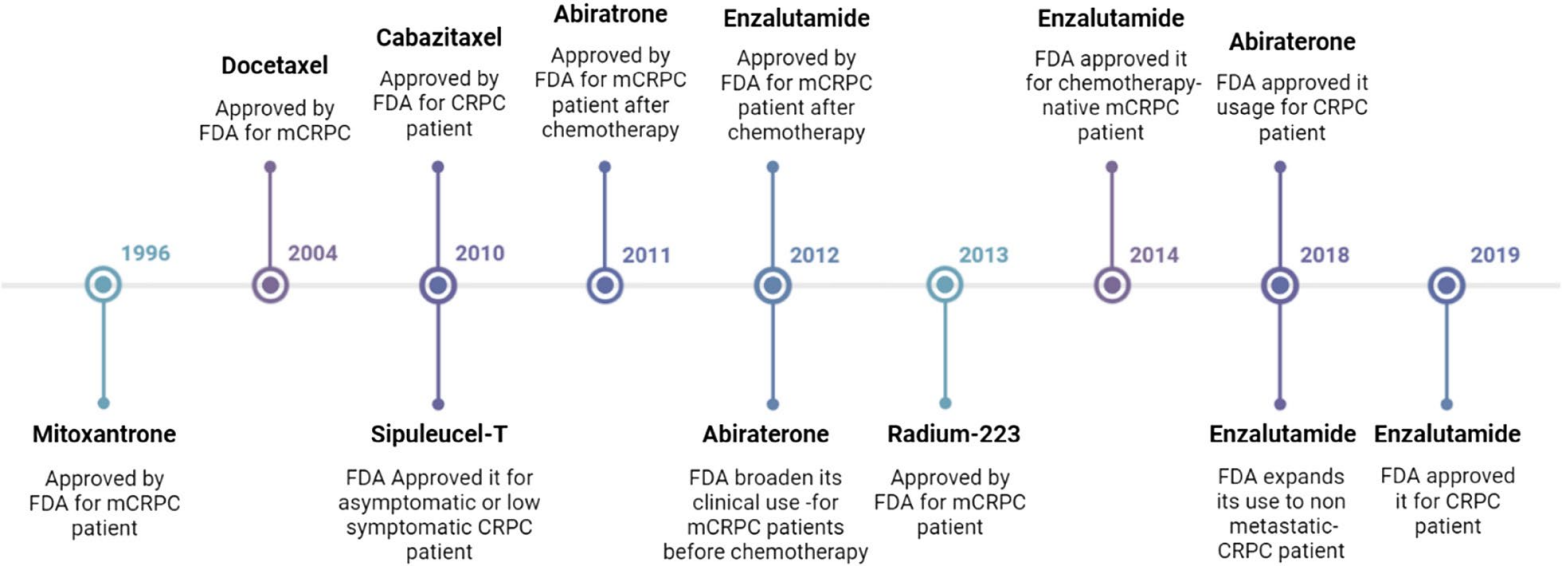
Timeline demonstrating evolution in the treatment regimen for PC

The primary methods for curing PC are pharmaceutical and surgical treatment. In recent years, inhibition of the androgen signaling pathway has emerged as a major therapeutic approach against tumors, where androgen levels are reduced by using hormones. Androgen deprivation therapy (ADT) is the name given to this treatment [26]. Hormone therapy such as ADT is highly effective in the treatment of metastatic hormone-sensitive PC (mHSPC), which leads to form metastatic castration-resistant PC (mCRPC) [27]. The approved drugs used for ADT are abiraterone acetate and enzalutamide. The drugs used in chemotherapy include docetaxel, cabazitaxel, mitoxantrone, and radium-223, which are radioisotopes used for cancer treatment [28]. The drugs got approval from United States Food and Drug Administration (US FDA) for the medication of PC has been listed in Table 1. The timeline for the development of these drugs and their respective approval year has been presented in Fig. 1. Surgical treatments are very successful in treating localized PC [29]. However, survivor of PC can have an adverse impact on the quality of life (QoL) of the survivor [30]. The most likely observed problem is depression [31] and many experiences of physical and sexual dysfunction [32].

**Table 1:** Different classes of drugs presently used to cure PC

Drug class	Representing drug	Anticancer: mode of action	Administration of drugs	Side effects of the drug	References
Taxane derivatives (Antimicrotubular agent)	Docetaxel	Docetaxel is a microtubule stabilizing taxane that inhibits microtubular assembly in cancer cells	75 mg/m^2^ once in every three weeks for six cycles, it is injected intravenously	Major side effects are nausea, vomiting, alopecia, neutropenia, fluid retention, gastrointestinal complications	[33, 34]
	Cabazitaxel	Cabazitaxel is a novel taxane-based chemo drug that blocks the mitosis of cancer cells at the metaphase anaphase transition by inhibiting the assembly of tubulin and induction of apoptosis	Used to treat advance stage prostate cancer, 20–25 mg/m^2^ is injected intravenously once every three weeks for six cycles	Side effects reported are neutropenic complications, fatigue, weakness, and diarrhea	[35, 36]
Anthracenedione derivatives (Alkaloids-cytotoxic agents)	Mitoxantrone	Mitoxantrone is a synthetic anthracenedione that has a variety of modes of action as it suppresses the T cells, B cells, and macrophages proliferation. It affects antigen presentation and reduces the inflammatory cytokines secretion. It is also an inhibitor of topoisomerase	The recommended dose is 12 mg/m^2^ intravenous infusion every three weeks	Cardiomyopathy, reduction in WBC count, discoloration of urine, and bleeding issues. Other common side effects are nausea, hair loss, and urine infections	[37, 38]
Androstane derivatives (Cytochrome P450 17A1 inhibitor)	Abiraterone acetate	Abiraterone is an androgen biosynthesis inhibitor that inhibits the 3-β hydroxysteroid dehydrogenase (3βHSD) mediated conversion of DHEA to androgen	It is orally administered 1000 mg daily with 10 mg of prednisone	Increased levels of mineralocorticoids lead to increased levels of k^+^ and hypertension. This issue is managed with the use of amiloride along with abiraterone	[39, 40]
Phenyl imidazolidine derivatives (Androgen receptor antagonist)	Enzalutamide	Enzalutamide is a second-generation antiandrogen that primarily targets the androgen signaling pathway. It is an AR blocker, so biosynthesis of androgen is reduced	It is orally administered at 160 mg daily	Fatigue, diarrhea, hot flushes, back pain, and hypertension	[41, 42]
Radiopharmaceutical agent	Radium-223	Radium-223 is an α-emitting radionucleotide that targets bone metastases in mCRPC patients	Four weekly intravenous infusions of 55 kBq/kg are administered for six cycles	Blood-related disorders like thrombocytopenia, anemia, and leukopenia have been reported. Some common side effects include fatigue and ostealgia	[43]
Therapeutic vaccines	Sipuleucel-T	Sipuleucel-T is an autologous cellular immunological agent	It is a cancer vaccine administered through leukapheresis. The infusion is given three times in a gap of two weeks	Arrhythmia, fever, pain due to blood clots, swelling, and chest pain	[44]

This review intends to provide an overview of PC, considering aspects such as the risk factors, clinical representations, different methods of diagnosis, treatment, and management, and the QoL of patients with PC. As PC has recently emerged as a global burden, extensive research and studies are required to better understand this disease so that novel diagnostic and therapeutic approaches can be identified to reduce mortality and improve patient QoL.

## Epidemiology

PC has always been the most common malignancy in men in the past few decades. In 2020, there were 1.41 million new PC cases globally, accounting for 7.3% of all cancer cases [2]. In 2021, there are 248,530 new PC cases reported and a sum of 34,130 deaths [45]. Furthermore, by 2040, these trends are estimated to increase to 2.43 million new cases and 740,000 deaths worldwide [46]. The Caribbean, Western and Northern Europe, North America, Australia, New Zealand, and Southern Africa had the highest incidence rates. At the same time, Northern Africa and Asia had the lowest incidence rates [2]. A major contributor to these varying PC incidence rates is variations in diagnostic practice. With the introduction of PSA screening in 1990s, a remarkable rise in PC incidence rates was noted in the United States, Australia, and Canada [3]. According to an investigation, one in every eight men has a risk of developing PC [45], with increasing age. For men below 50 years of age, the probability of developing PC has been reported to be low in comparison to those aged 50–59 and 60–69 years [47].

Approximately one in every 41 men dies because of PC [45]. The mortality trends did not show as much variation as the incidence rates. The highest rate of mortality are observed in regions such as sub-Saharan Africa, the Caribbean, and Micronesia/Polynesia [2]. In contrast, low mortality has been observed in South Central and Eastern Asia [3]. Currently, there are more than 3.2 million survivors of PC in the United States [48]. There has been a remarkable decline in PC incidence rates in the last decade. Due to concerns regarding the over diagnosis and over treatment of PC.

## Major risk factors

Major risk factors associated with the incidence of PC includes the aging, race, family history, and genetic factors. Besides these the lifestyle factors such as diet, obesity, and smoking has also been reported to be associated with the prevalence of PC [49].

### Age

PC is commonly diagnosed in men above 60 years of age. The United States Preventative Services Task Force (USPSTF) issued Grade ‘D’ recommendations for discouraging PSA use for men above 75 years of age in 2008 and all men in 2012. Subsequently, PSA screening was reduced by 25–30%, resulting in a significant decline in incidence rate of PC in the United States. In 2018, the USPSTF issued a new recommendation, stating periodic PSA screening for men aged 55–69 years. In contrast, PSA screening is discouraged in aged men above 70 years [50]. The median age of diagnosis has been reported to be 67 years, with more than 30% of deaths reported in those aged 75–84 years [48].

### Race and ethnicity

Racial disparity has been observed in PC. African black men have a higher incidence rate than white men [51]. A report from the United States of America showed that African Americans have 58% more incidence and 144% more mortality than white men of European ancestry. In contrast, Hispanics have been reported to 14% lower incidence and 17% lower mortality than white men [52]. The reason for this disparity in PC is socioeconomic conditions and biological factors [53]. African American men have chromosome 8q24, which has been reported to be associated with an increased risk of PC. They also have tumor suppressor genes, such as EphB2, which increase cancer risk [54, 55].

### Family history and genetic factors

Family history plays a role in the development of PC. The risk of developing PC is higher in men who have a first-degree relative with PC. Furthermore, the risk is higher if the relative is a brother [56]. Genetic factors contribute to nearly 40% of the risk of developing PC. Mutations in BRCA1 and BRCA2 increase the risk of PC. A mutation in the BRCA2 gene confers 8.6 times increased risk of PC in men aged < 65 years [57, 58]. Other rare mutations in PC include HOXB13, NBS1, and CHEK2 [59].

### Lifestyle factors

Major lifestyle factors that influence the development of PC include diet, obesity, and smoking.

### Diet and obesity

Diet plays a vital role in the incidence of PC, and there are certain food products that have a higher risk for PC, such as saturated animal fat, red meat, and dairy proteins. However, some dietary products decrease the development of cancer, such as soya, green tea, tomatoes, and lycopene [4, 60]. According to a survey, a sedentary lifestyle is related to increased PSA levels [61]. Some meta-analyses have reported a clear association between obesity and an enhanced incidence of PC independent of body mass index increases [62]. Obesity has been reported to be associated with a high risk of aggressive PC. The involvement of certain biological mechanisms, such as the development of insulin resistance due to physical inactivity, sex steroid hormones, and changes in metabolism, supports an association between obesity and PC [63].

## Smoking

Smoking has been associated with the incidence of all types of cancers. Several reports suggested that smoking is associated with a greater number of mortalities due to PC. It also depends on the number of cigarettes smoked per day [64]. A California report stated that the rate of smoking is declining by 3.5% per year, resulting in a 2.5% decrease in PC death, suggesting that smoking reduction is beneficial for the decline in cancer mortality [65].

### Novel diagnostic approaches

The traditionally used PSA test for the screening and diagnosis of PC has various limitations, including its low specificity. These challenges have led to over diagnosis and over treatment of low-risk PC patients. Thus, new diagnostic methods that are cost-efficient and non-invasive are needed to differentiate between aggressive and slow PC and to decrease the performance of excessive biopsies [66].

In the last decade, an increasing knowledge of the genetics and molecular biology of PC has led to the identification of several biomarkers that overcome the existing limitations of PSA. These newer biomarker-based methods with more specificity have been shown to be significantly better. Thus, these biomarker-based methods can help reduce the overtreatment and overdiagnosis of PC [66]. These biomarkers are used at various stages of decision making, including screening, after a positive biopsy for risk stratification, after a negative biopsy to determine whether to consider a repeat biopsy, and to monitor prognosis after treatment or in those suspected of recurrence to determine whether additional treatment is required. Despite these potential benefits of biomarkers, most are not currently used in clinical practice because there is a lack of clinically significant support validating their utility and benefits [67]. Nevertheless, we will now discuss some biomarker-based diagnostic tests that have demonstrated clinical significance.

### Novel biomarker-based diagnostic approaches

Biomarkers are used for various reasons, such as screening, diagnosis, risk stratification, and prognosis. A concise overview of various biomarker-based tests used in the diagnosis of PC can be seen in Table 2.

**Table 2:** Clinically significant different biomarkers for the diagnosis of PC

Test	Company	Biomaterial	Biomarker details	Clinical utility	Test outcome	Certification	References
PHI	Beckman Coulter Inc	Blood serum	Free PSA (fPSA), total PSA (tPSA), and [-2] proPSA	Initial biopsy and rebiopsy	Differentiates benign conditions and malignant prostate cancers Decreases unnecessary biopsies	US FDA	[68]
4K score	OPKO Health Inc	Blood plasma	Free PSA (fPSA), total PSA (tPSA), intact PSA (iPSA), and human kallikrein 2 (hK2)	Initial biopsy and rebiopsy	Differentiates high-grade and indolent prostate cancer Decreases unnecessary biopsies	CLIA	[69]
PCA3	Hologic Inc	Post DRE urine	PSA mRNA, lncRNA PCA3	Rebiopsy	Decreases unnecessary biopsies	US FDA	[70]
Exo-Dx	Exosome Diagnostics	Urine	Exosomal mRNA (PCA3, ERG, and SPDEF)	Initial biopsy and rebiopsy	Differentiates high-grade and indolent prostate cancer Decreases unnecessary biopsies	CLIA	[71]
SelectMDx	MDxHealth	Post DRE first void urine	DLX1, KLK3, HOXC6, mRNA and PSA	Initial biopsy and rebiopsy	Differentiates high-grade and indolent prostate cancer. Decreases unnecessary biopsies	CLIA	[72]
TMPRSS2-ERG	Blood tissue and urine	Post DRE urine	Fusion gene TMPRSS2-ERG	Initial biopsy	Differentiates high-grade and indolent prostate cancer Decreases unnecessary biopsies	No	[73]
Mi-Prostate Score	Michigan Labs	Post DRE first void urine	PCA3 and TMPRSS2-ERG mRNA, tPSA	Initial biopsy	Differentiates high-grade and indolent prostate cancer Decreases unnecessary biopsies	CLIA	[74]
ConfirmMDx	MDx Health	Tissue	Hypermethylation of genes- GSTP1, APC and RASSF1, PSA	Rebiopsy	Decreases unnecessary biopsies	CLIA	[75]

CLIA: Clinical Laboratory Improvement Amendments under Center of Medicare and Medicaid Services

## Serum-based biomarkers

### Prostate health index (PHI)

The PHI test is a serum-based analysis, developed by Beckman Coulter Inc. and the National Cancer Institute-Early Detection Research Network. The biomarkers included in the PHI are [-2] proPSA, free PSA (fPSA), and total PSA (tPSA). These individual values are then subjected to the formula [-2] proPSA/fPSA × √tPSA to generate a score that can differentiate between benign and malignant PCs, decreasing the performance of unnecessary biopsies [68]. Following this equation, high-risk patients are those with increased tPSA and [-2] proPSA levels and decreased fPSA levels. Thus, owing to the high risk of aggressive PC, patients with high PHI scores are suggested to undergo less invasive biopsies [76]. In 2012, the US FDA authorized the commercial use of PHI for patients > 50 years of age with PSA levels of 4–10 ng/mL and negative DRE reports [68].

Catalona et al. conducted a multicenter trial in the United States on the clinical significance of PHI in 892 men considered for biopsy with total PSA between 2 and 10 ng/mL and normal DRE. Their results indicated that PHI had significantly higher specificity with 80–95% sensitivity than PSA and %fPSA alone. The AUC values of PHI were 0.70, whereas PSA and % fPSA have AUCs of 0.53 and 0.65, respectively [77].

Another study of two cohort groups, including 561 and 395 men with no prior biopsy, demonstrated that PHI could predict the possibility of high-grade PC (AUC -0.81 and 0.78). PHI had a specificity of 36% when the sensitivity settings were at 95%, which is comparatively high with the specificity of fPSA and tPSA, i.e., 19.4% and 17.2%, respectively. A PHI threshold value of ≥ 24 was optimum at 95% sensitivity, with a 36% reduction in unnecessary biopsies and a few missed biopsies [78]. White et al. performed a study including two groups, with 506 men undergoing the PHI test and 683 men as controls. In this study, 73% of patient management was influenced by the PHI score, where ≥ 36 was considered the threshold. A notable reduction in unnecessary biopsies was observed among men that underwent PHI tests compared to controls (36.4% vs. 60.3%) [79].

### 4K score

The 4K score is a blood-based test, developed by OPKO Health Miami, FL, USA. Patients with abnormal PSA or DRE levels undergo a 4K score test to decide whether an initial biopsy is needed and patients for whom repeat biopsy is being considered. The possible candidates for this test are men with a family history of PC, but any man above 35 years of age, curious about his risk, can have this test [76].

The 4K score utilizes four levels of kallikreins- fPSA, iPSA, tPSA, and human kallikrein 2 (hK2) along with the patient data, such as DRE, age, and initial biopsy outcomes in a specific algorithm, providing results in the form of percentages from 0 to 100%. This percentage forecasts the probability of significant high-grade PC before biopsy. The 4K score can differentiate between patients with aggressive and slow PC. The patients with a Gleason score > 7 from those with a Gleason score < 7 have been considered as an aggressive in nature. The test can evaluate the threat of distant metastasis within 20 years of performing the test [69].

To validate the clinical utility and significance of 4K scores, Parekh et al. performed a trial in the United States that included 1021 men considered for biopsy, where 23% of men were found to have a Gleason score ≥ 7 PC. The 4K score demonstrated better accuracy than the Prostate Cancer Prevention Trial Risk Calculator 2.0 (PCPT-RC). The overall reduction in biopsies was reported to range between 30 and 58% depending on different thresholds, with only 1.3–4.7% cases of late diagnosis. When using a ≥ 6% probability of having a Gleason score ≥ 7 as the threshold for performing a biopsy, there would be a 30% reduction in biopsies with only 1.3% delayed cases. Similarly, when ≥ 9% and ≥ 15% is the threshold, the reduction in biopsies is 43% and 58%, with 2.4% and 4.7% delayed cases, respectively [80].

A case–control study of multi-ethnic groups involving 1,667 PC incidents and 691 controls with PSA levels of ≥ 2 ng/mL was conducted. The multi-ethnic groups included were native Hawaiians, White men, Latinos, African Americans, and Japanese. The outcomes demonstrated that among all ethnic groups, the 4K score has a higher ability to differentiate overall and aggressive PC compared to tPSA and tPSA + fPSA. Thus, the 4K score appears to be a better alternative in a multi-ethnic population, providing evidence of its more comprehensive utility [81]. Various studies have found that the 4K score test decreases the cost while simultaneously improving the quality of patient care [82, 83]. A comparative study of the PHI and 4K score showed that both increased discrimination while predicting high-grade PC and PC [84].

## Urine-based biomarkers

### Prostate cancer antigen 3 (PCA3)

The PCA3 gene codes for long non-coding ribonucleic acid (RNA), previously known as DD3. In more than 90% of PC cases, the PCA3 gene is 60–100 folds overexpressed compared to the normal tissues [85]. The PCA3 test was made commercially available by Hologic Inc. It is a non-invasive method and utilizes the amount of PCA3 and mRNA of PSA found in urine collected after DRE [70]. The levels of PCA3 and mRNA of PSA were quantified using quantitative real-time polymerase chain reaction (qPCR). This information was substituted in the formula PCA3 mRNA/ mRNA of PSA × 1000, generating a PCA3 score that predicts the probability of PC, decreasing the performance of unnecessary biopsies. A score of PCA3 (≥ 25) suggests a high probability of PC, whereas a score of < 25 is considered a low probability of PC [70]. Various studies have suggested a different threshold for PCA3 scores. Thus, the threshold ranges between 25 and 35 accordingly, and the reduction in unnecessary biopsies also changes from 37% to 77.1% [72]. The optimum threshold value for PCA3 remains contentious.

Merola et al. have performed a clinical study involving 407 men to assess the accuracy of PCA3 compared to total PSA and f/t PSA. They demonstrated that PCA3 surpassed the f/t PSA performance. When the cutoff score was 35, PCA3 was found to have better sensitivity (94.9%) and specificity (60.1%) compared to a cutoff score of 20 [85]. According to a meta-analysis of nine studies, PCA3 has an AUC of 0.734, with a significant sensitivity and specificity by 69% and 65%, respectively. This study showed that a cutoff score of 35 had better clinical accuracy and applicability than other values [86].

### Exo-Dx (Prostate IntelliScore) (EPI)

Exo-Dx (Prostate IntelliScore) is a urine-based test. Exosomes are double-layered small vesicles that contain various cellular proteins produced by cells. This test measures exosome expression in urine by quantifying ERG and mRNA of PCA3 normalized to the SAM pointed domain containing ETS transcription factor (SPDEF) [87]. The results from this test are expressed as an EPI score ranging from 0 to 100 [71]. This test measures exosome gene expression in urine and does not require prostatic massage or pre-DRE for sample collection [87]. Based on the guidelines of National Comprehensive Cancer Network, this test can be useful in distinguishing the low-grade PC from high-grade PC in patients of more than 50 years of age with PSA levels of 2–10 ng/mL [87]. In a clinical trial study conducted on 503 men with an average age of 64 years and PSA level of 5.4 ng/mL has reported a reduction in total number of biopsies by 20%, unnecessary biopsy by 26%, and the number of missed biopsies by 7% with a negative predictive value (NPV) of 89% [88].

### SelectMDx

SelectMDx is a urine-based test developed by MDx Health, Inc. Usually, a urine sample is taken after DRE. This quantifies the mRNA levels of HOXC6 and DLX1, which are considered as a biomarker gene [89]. Various clinical data, the PSA levels, DRE results, PSA density, and history of PC were also considered while calculating the score [72]. In a study with two cohorts of 519 and 386 patients, respectively, SelectMDx demonstrated its accuracy in predicting PC with the Gleason score of 7, an AUC of 0.86, and a negative predictive threshold of 98%. They also suggested that this test could reduce unnecessary biopsies by 53% and overall biopsies by 42% [90]. A study done by Haese et al. in European countries included 1955 men with PSA levels less than 10 ng/mg and tested their urine samples. At a sensitivity of 47%, SelectMDx had a specificity of 47% and an AUC score of 0.85. The NPV was 95%. The AUC values of this study were compared with those of PCPT-RC, which had an AUC of 0.76. Thus, the test outperformed the PCPT-RC results [91]. A study found that the clinical use of SelectMDx is cost-efficient in European countries [92].

### Mi-Prostate Score (MiPS)

The MiPS was discovered by the University of Michigan, Michigan Labs. Urine samples were collected after a DRE or prostatic massage. MiPS measures the mRNA expression levels of the TMPRSS-ERG fusion gene, PCA3, and tPSA [93, 94]. This test is considered for patients undergoing initial biopsy, and the results predict the possibility of high-grade PC [74]. Some studies have found that the combination of PCA3 and TMPRSS2-ERG enhances diagnostic capabilities [95].

Sanda et al. conducted a multicenter study to analyze the clinical applicability of the MiPS. In a validation group of 561 men with an average age of 62 years, they found that MiPS enhanced the specificity from 17 to 33%, distinguishing high-grade PC with a sensitivity of 93%. They also observed a 42% reduction in unnecessary biopsies using the MiPS test before biopsy [96]. A validation study by Tomlins et al. demonstrated that MiPS outperforms PSA alone in diagnosis. When detecting PC, the AUC value of MiPS was 0.751, whereas the PSA AUC value was 0.585. Similarly, while detecting clinically significant high-grade PC, MiPS and PSA have AUC values of 0.772 and 0.651, respectively [97].

## TMPRSS2-ERG fusion gene test

TMPRSS2-ERG (androgen-related transmembrane protease serine 2) and ERG (ETS-related gene) are present on chromosome 21. In 2005, TMPRSS2-ERG was found to be fused in 40–80% of PC cases. The TMPRSS2-ERG test gives a score that can predict the possibility of high-grade PC. This score is calculated using the formula: (TMPRSS2-ERG mRNA/PSA mRNA) × 100,000 [73]. TMPRSS2-ERG often combines with PCA3 to improve its predictability. The predictive value of this test is still under investigation [98].

## Tissue-based biomarkers

### ConfirmMDx

Initially, there were 20–30% chances of false-negative reports in the histological test of prostate biopsies. Thus, patients at potential risk of PC needed to undergo repeat biopsies, affecting low-risk patients. Thus, to avoid unnecessary biopsies in these patients, ConfirmMDx was designed. ConfirmMDx is a biopsy-based test that requires tissue samples to provide results. MDx Health Inc. developed this test. This epigenetic test utilizes methylation-specific PCR to evaluate the DNA hypermethylation of APC, GSTP1, and RASSF1. This test can histologically distinguish between normal and cancerous cells. Patients with negative ConfirmMDx results were found to have a < 5% lower probability of a rebiopsy, which is ten times decrease in the initial rates [75].

A multicentre study in the United States which involved 350 men who underwent for the repeated biopsy after a previous negative biopsy underwent the ConfirmMDx test within 24 months of the biopsy. The results showed an NPV of 88% and confirmed its predictive value in multivariate analysis. Therefore, unnecessary repeat biopsies can be avoided [99]. The MALTOC trial included 498 men with negative biopsies who underwent the ConfirmMDx test within 30 months of the previous biopsy. This trial showed an NPV of 90%, and the multivariate analysis showed its significance in predicting outcomes [100]. In a study done by Yonover et al. in the United States, 605 men with an average age of 64 years, average PSA of 6.8 ng/mL, and a negative biopsy report underwent the ConfirmMDx test within ten months of the previous biopsy. They found that the test significantly impacted clinical decision making in rebiopsy settings [101].

## Emerging biomarkers for the diagnosis and prognosis of PC

Various potential molecular biomarkers for PC diagnosis and prognosis are still developing. Some of them are under investigation such as circulating tumor cells (CTCs), PTEN, androgen receptor variants, long non-coding RNAs such as HOX transcript antisense intergenic RNA [102, 103], SChLAP1 and MaLAT-1, and several miRNAs such as miRNA-141 and miRNA-301a. However, most of these biomarkers are yet to be approved for clinical use [104]. Feng et al. observed that spindle and kinetochore-associated complex subunit 3 (SKA3) is highly upregulated in PC cells compared to normal cells. Further, PC patients with higher expression of SKA3 are associated with an increased risk of rapid progression to metastasis [105].

## Biomarkers of liquid biopsy: new edge technology for PC patients

Liquid biopsy is a non-invasive tool used for the diagnosis and management of PC patients. It is a more advanced approach for early detection and monitoring of PC. It utilizes blood, urine, and other body fluids as samples and facilitates different targeted therapies, and probability of resistance to therapies [106]. It analyses samples in real-time identifying and enumerating circulation tumor cells, cell-free DNA (cfDNA), circulating tumor DNA/RNA (ctDNA/RNA), and extracellular vesicles [107].

CTCs are cells shredded from primary or a metastatic tumor mass, circulating in the blood. CTCs are epithelial cell adhesion/activating molecule (EpCAM)-positive cells, which are used as a biomarker for the identification and enumeration of PC [108]. EpCAM is a transmembrane glycoprotein, its expression is upregulated in cancer cells. In cancer cells, it is responsible for cell adhesion, proliferation, angiogenesis, stemness, chemotherapy resistance, and epithelial to mesenchymal transition. Thus, EpCAM is a diagnostic and prognostic biomarker as well as a potential target for more precision therapy [108, 109]. The EpCAM-dependent CTC test has been the only US FDA approved technology to be used for clinical application [110]. CTCs of more than 5/7.5 ml of blood are considered unfavourable with shorter PFI and overall survival (OS) [107]. EpCAM independent CTC capture methods are also being developed such as epithelial immunospot (EPISPOT). It is an antibody-based method for quantification of live CTCs by detection of CTCs which are capable of secreting proteins such as cathepsin D, MUC1 and CK19 [111, 112]. For PC cells, PSA and FGF2 are target proteins for identification [113]. Another CTC biomarker under clinical investigation is AR-V7 [114].

## Multiparametric magnetic resonance imaging (mpMRI)

mpMRI has enhanced the diagnosis, reducing unnecessary biopsies and improvised risk stratification system for prostate cancer patients [115]. mpMRI is performed when a person shows abnormal screening. If lesions (PI-RADS ≥ 3) appeared in MRI, then patient is recommended for targeted biopsy or systematic biopsy [115]. In 2019, the lesions of a clinically significant PC ranges from PI-RADS 1 to PI-RADS 5. Various lesions are graded as PI-RADS 3 or 4 characterised by mild to high chances of converting then into tumour in future [116].

## Therapeutic strategies

PC is one of the leading cancers reported worldwide, and despite various advances in medical science. The treatment of PC still needs further improvement. Major therapeutic approaches for PC include surgical treatment, radiotherapy, chemotherapy, and hormone therapies [117]. The correct approach for the treatment of PC depends on whether we are trying to cure the disease or control certain symptoms. It also depends on an individual’s risk of death from other causes and the life expectancy. In nearly 80–90% of PC cases, increased androgen activity is detected in the initial stage of the disease. Hence, inhibition of the AR and reduction in androgen levels are the cornerstones of ADT. Therefore, ADT remained the first line of treatment for men with PC. ADT is variable as 20–30% of patients show tumor recurrence and become castration resistance, so a metastatic hormone naive tumor becomes mCRPC [118]. Men with localized PC have three treatment options: close monitoring, surgical treatment, and radiotherapy. Patients of metastatic PC has been reported to be treated with chemo-hormonal therapy such as docetaxel novel hormone therapy and cell-based cancer immunotherapy [119]. There is no specific sequential order of therapy for patients with PC because of rapid changes in treatment options and the approval of new drugs. We will now discuss some of the therapeutic options widely used by physicians to treat patients with PC.

## Chemotherapy

Drugs approved for therapy include docetaxel, cabazitaxel, mitoxantrone, and bicalutamide (first-generation antiandrogens).

### Docetaxel

**Fig. 2:**
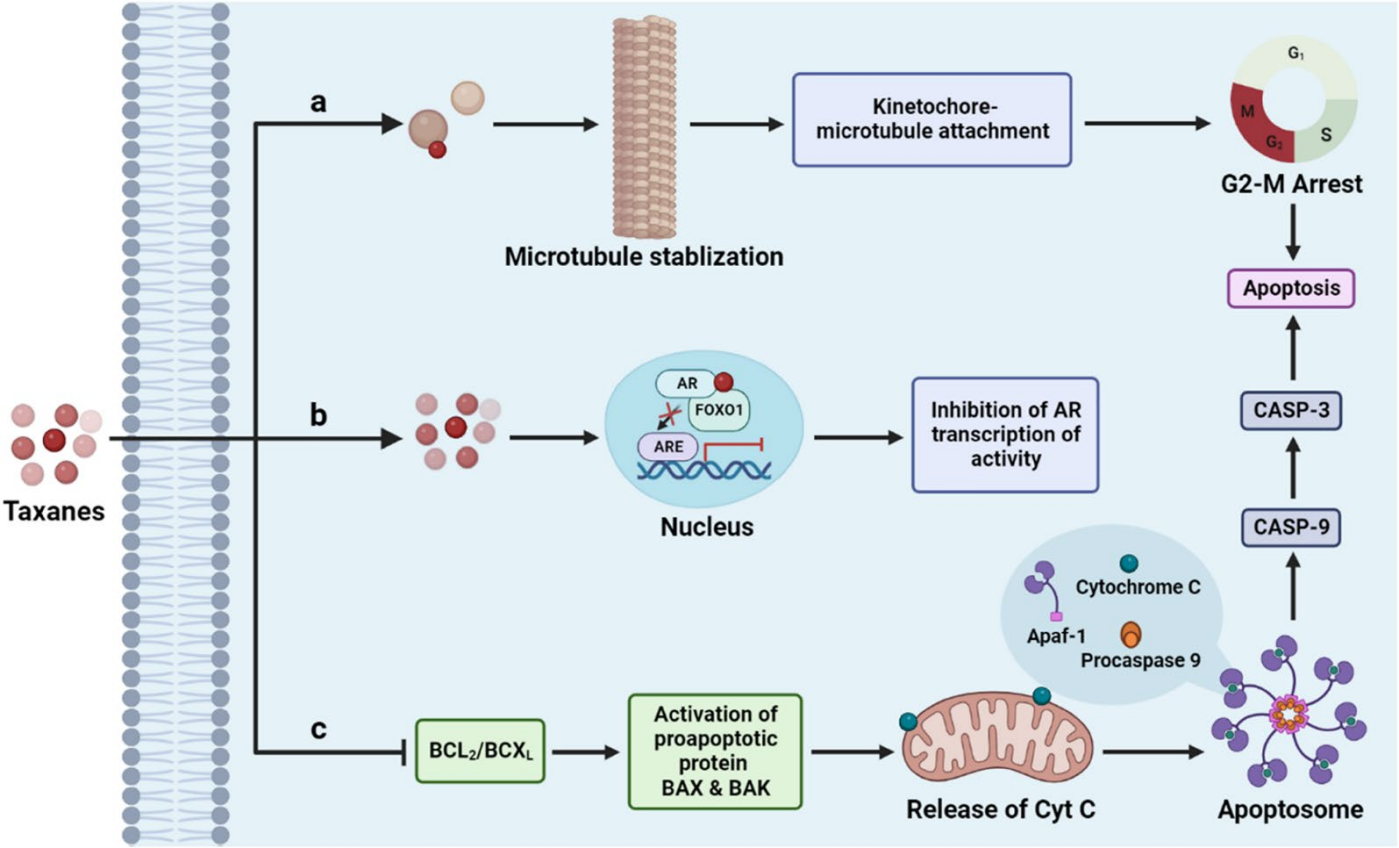
Mechanism of action of Taxanes: **a** Taxane derivatives bind to β tubulin which leads to microtubule stabilization, this inhibits the proper assembly of microtubules and inhibits G2-M transition and apoptosis. **b** Taxanes are able to inhibit AR activity by FOXO1 mediated inhibition of AR transcriptional activity. **c** Taxanes inhibit antiapoptotic proteins (BCL2, and BCL-XL) and promotes activation of proapoptotic proteins (BAX, and BAK) leads to the release of cytochrome c that activates intrinsic apoptotic pathway, which leads to cell death (AR: Androgen Receptor and ARE: Androgen Receptor Element)

Docetaxel is a taxane-based chemotherapeutic drug used to treat PC. It shows anticancer activity by inhibiting microtubule assembly during mitosis and interphase, leading to cell death (Fig. 2), and is thought to have some anti-androgenic properties [120]. Following several phase trials, this chemotherapeutic drug was the first found to increase OS of patients with PC. A multi-arm cohort study conducted on 593 patients, with the administration of ADT and docetaxel (75 mg/m^2^) after every three weeks and prednisone (10 mg/day) has showed a significant improvement in the OS compared with 1184 patients administered ADT alone. Therefore, this drug has an OS benefit when combined with another hormonal drug and steroids [121]. Docetaxel is usually administered intravenously once every three weeks for ten cycles. However, reducing the drug dose depends on the patient’s tolerability. Furthermore, like other chemotherapy drugs, it is also associated with certain side-effects, such as cytopenia, nausea, vomiting, and neutropenic sepsis [122].

### Cabazitaxel

Cabazitaxel is a US FDA approved semisynthetic compound. This compound is being used as a second line of therapeutic after docetaxel in patients with PC. This chemotherapeutic drug also has the same mode of action as docetaxel as it inhibits microtubule assembly (Fig. 2) [123]. It can overcome taxane resistance and shows anticancer activity in patients with post-docetaxel treatment and docetaxel-resistant cancers [124]. Cabazitaxel is administered via an intravenous infusion once every three weeks, and the standard dose is 25 mg/m^2^ fixed after studying various trials [125]. Detailed mechanism of action of taxanes like Docetaxel and Cabazitaxel is demonstrated in Fig. 2.

### Mitoxantrone

Mitoxantrone is a synthetic compound, used as a second-line chemotherapeutic drug for the treatment of PC. Mitoxantrone causes immunogenic cell death in PC cells by activating eukaryotic initiation factor 2 [126]. Retrospective analysis of data from various phase 3 trials of mitoxantrone revealed symptomatic improvement without any survival benefits in some patients, as well as adverse impacts, such as fatigue, shortness of breath, and pancytopenia [127].

## Novel hormone therapies

Novel hormone therapy, also known as androgen suppression therapy, is used to suppress the levels of androgen by targeting the androgen signaling pathway [128]. Overexpression of androgen is responsible for the progress of both mHSPC and mCRPC [129]. Some drugs that have regulatory approval include abiraterone and enzalutamide [130–132].

### Abiraterone

**Fig. 3:**
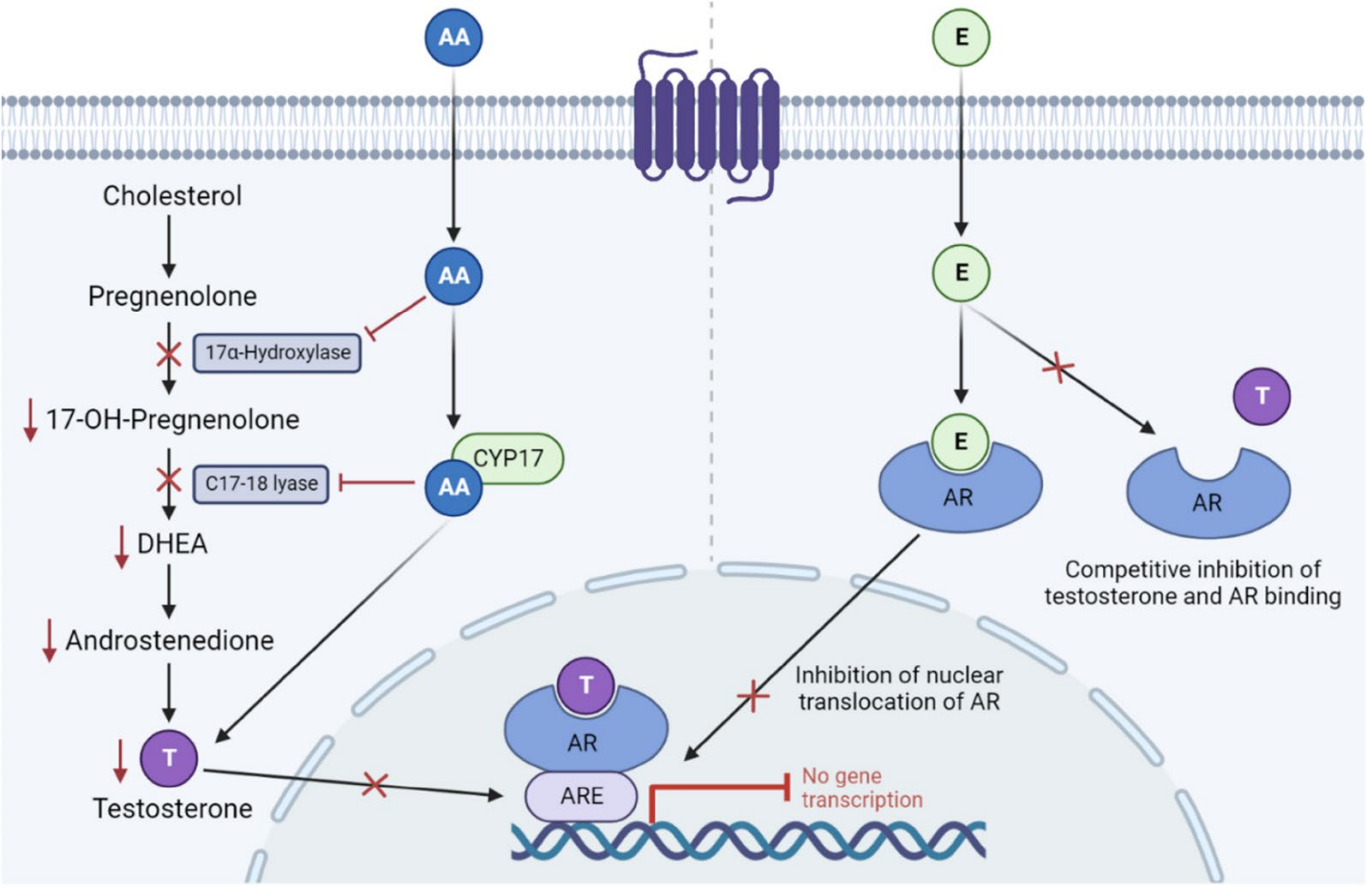
Mechanism of action of Abiraterone acetate and Enzalutamide. Abiraterone acetate inhibits the synthesis of androgen by blocking the action of 17α-hydroxylase and C 17, 20-lyase on CYP-17, leading to the inhibition in binding of testosterone to AR. Enzalutamide binds with AR and inhibits the binding of testosterone to AR. It also inhibits nuclear translocation of AR (AA: Abiraterone acetate, E: Enzalutamide, ARE: Androgen Receptor Element, AR: Androgen Receptor, T: testosterone, DHEA: Dehydroepiandrosterone)

Abiraterone acetate is an irreversible, selective inhibitor of cytochrome p450 17A1 (CYP17), which blocks the production of androgen in men with mCRPC [133–135]. Abiraterone, has been shown to provide a survival benefit and therefore approved for the treatment of mCRPC [136–138] by targeting androgen pathway (Fig. 3) [139]. Figure 3 shows precise mode of action of Abiraterone acetate and Enzalutamide as androgen suppression therapy. A random study conducted on 1,917 patients showed that abiraterone, ADT, and prednisone showed higher OS [140]. Abiraterone is orally administered at a dosage of 1000 mg/day and a low dose of prednisone has been reported to show fluid retention, hypertension, and hypokalemia with specific side effects such as increased levels of mineralocorticoids induced by the block of CYP17 [141].

### Enzalutamide

Enzalutamide is a second-generation antiandrogen therapeutic approved for the medication of CRPC. It is highly effective in men with non-metastatic CRPC and reduces the probability of metastasis and death by 71% [142]. Various phase 3 trials have shown that enzalutamide has anticancer activity and improves OS before and after chemotherapy. Enzalutamide is prescribed to be administered orally once at a dose of 160 mg/day. The most commonly observed side effects are gastrointestinal problems, fatigue, and hot flushes [143].

## Radiotherapy

Radiation therapy is generally used to cure the early stages of PC by treating locally advanced tumors and reducing the risk of metastasis. This therapy has been slowly evolving. Indeed, various radiotherapy techniques have been developed, such as image-guided radiotherapy, stereotactic ablative body radiotherapy, intensity-modulated radiotherapy, and volumetric modulated arc therapy brachytherapy. Despite significant improvements in radiotherapy, many PC patients still suffer from the recurrence of the disease, due to the low specificity of therapy. Increasing the radiation dose to achieve adequate anti-cancer effects can cause damage to the healthy tissues and result in adverse impacts. New techniques, such as proton and carbon ion therapy, maximize their effect on tumor cells while minimizing their effect on surrounding healthy cells [144]. Nearly 10–40% of patients show tumor recurrence after having radical prostatectomy; for them, salvage radiation therapy is very effective and allows control of diseases in 60–70% of cases [145]. Radiation therapy can have certain radio-induced side effects in patients, such as radiation proctitis. The correct positioning of patients and set up verifications can help to prevent and/or reduce the risk of radiation proctitis [146]. Radical prostatectomy remains the first treatment option for localized PC and reduces the risk of mortality. It provides better survival chances than radiotherapy in patients with a primary tumor [147]. Major prostatectomies include open prostatectomy, laparoscopic prostatectomy, and TURP.

Radium-223 dichloride, also called alpha radio, is a radiopharmaceutical that emits alpha particles that selectively target bone metastases [148]. Radium-223 is specifically used to treat patients with mCRPC with bone metastases to induce irreversible double-strand breaks in DNA, leading to tumor cell death [149]. It is usually administered through intravenous infusion for four weeks with six cycles. The most commonly reported side effects include bone pain, fatigue, gastrointestinal disturbances, hematological toxicity, thrombocytopenia, and leukopenia, which affect the adjacent bone marrow [150].

## Phototherapy

Phototherapy (PT) uses the materials which can absorb the electromagnetic energy and transform it into thermal energy to induce apoptosis in cancer cells [151]. This approach eliminates the risk of infection at the time of surgery and get rid of the side effects of chemotherapy. Near-infrared (800–1350 nm) light is primarily employed for the PT. The materials which have the ability to transform near-infrared radiation into temperature has been used for the PT and are recognized as the photothermal agents [151–153]. PT has been blended with other therapeutic regimens such as immunotherapy or chemotherapy for enhancing the therapeutic impact against tumors [154]. Photodynamic therapy (PDT) is also an approach for the treatment of cancer which promotes the generation of reactive oxygen species in cancer cells leading to the induction of apoptosis in cancer cells [155, 156]. Polymer-coated metal nanostructures have been used for the PDT. It has also been employed for improving the efficacy of radiotherapy in suppressing the liver cancer [157]. Silver-gold hollow nano shells with mesoporous silica nanoparticle have been utilized for the PDT in the experimental PC cells [158]. In another study, a hybrid nanoparticles of Au-alendronate were made for combined PDT-chemotherapy of PC [159]. The PDT not only enhanced the impact of chemotherapy in suppressing PC, but also decreased the dose of the chemotherapy needed for the treatment of PC [160–162].

## Immunotherapy

Immunotherapy (IT) has moved the therapy concept of different tumors/cancers in clinical scenarios [163, 164]. However, the clear impact in the suppression of PC has not been reported yet [165]. PC remained as a tumor with predominantly immunosuppressive components, like regulatory T cells (Tregs) and transforming growth factor-β (TGF-β). Although tumor cells has been reported to express certain marker antigens, such as PSMA and PSA [166]. There are several immunotherapies reported to optimize the available treatment strategies.

Anticancer agents are designed to join with high selectivity of monoclonal antibodies to form antibody–drug conjugates such as Sacituzumab govitecan, trastuzumab deruxtecan targeting Trop2, and HER2. It has the capability to directly carry cytotoxic drugs to tumor [167]. The treatment of PC with immunomodulatory drugs includes both active as well as passive approaches. The active approach includes vaccines which are designed with the intent to stimulate an adaptative immune response through presentation of an antigen [165]. The passive approach includes the administration of highly specific monoclonal antibodies for tumor-associated antigens (TAAs) and tumor-specific antigens (TSAs). The efficacy of anticancer vaccines of PC can be analyzed using its specific biological markers or features, which include an early diagnosis of disease recurrences, slow growth, and a series of TAAs (PSA, and PSMA), TSAs [168], prostate stem cell antigen (PSCA), prostate acid phosphatase (PAP), PCA-3 antigen, six-transmembrane epithelial antigens of prostate (STEAP), and mucin-1 [169]. It has reported that vaccines can be used along with the other therapeutics such as second-generation hormonal treatments (docetaxel, and radiotherapy) [170]. Vaccine-based treatments can be categorized into two groups such as cell-based and viral vector-based vaccines [168, 171, 172].

Chimeric antigen receptor (CAR) T-cells is a recently advancing approach of immunotherapy for the treatment of solid tumors where antibody fragments are used along with T cells specific against TSAs [173, 174]. CAR T-cell therapy have been reported to show a remarkable success in B cell hematological malignancy [175–178]. There are several newly emerging TAAs under investigation such as immune checkpoint B7-H3 (CD276), Mucin-1, IL-6 receptor (CD126), Lewis-y antigen, STEAP-1 [179]. Major challenges faced by this therapy are manufacturing of CAR T-cells, direct attack on normal tissues that share expression of the TAAs called On-Target Off-Tumor toxicity, and cytokine toxicity [180–182]. IT has emerged as a potential treatment regimen for mCRPC. It can significantly improve progression-free survival and overall response rate especially in immune checkpoint inhibitors treatment. However, they cannot improve the OS [183].

## Cell-based vaccines

### Sipuleucel-T

**Fig. 4:**
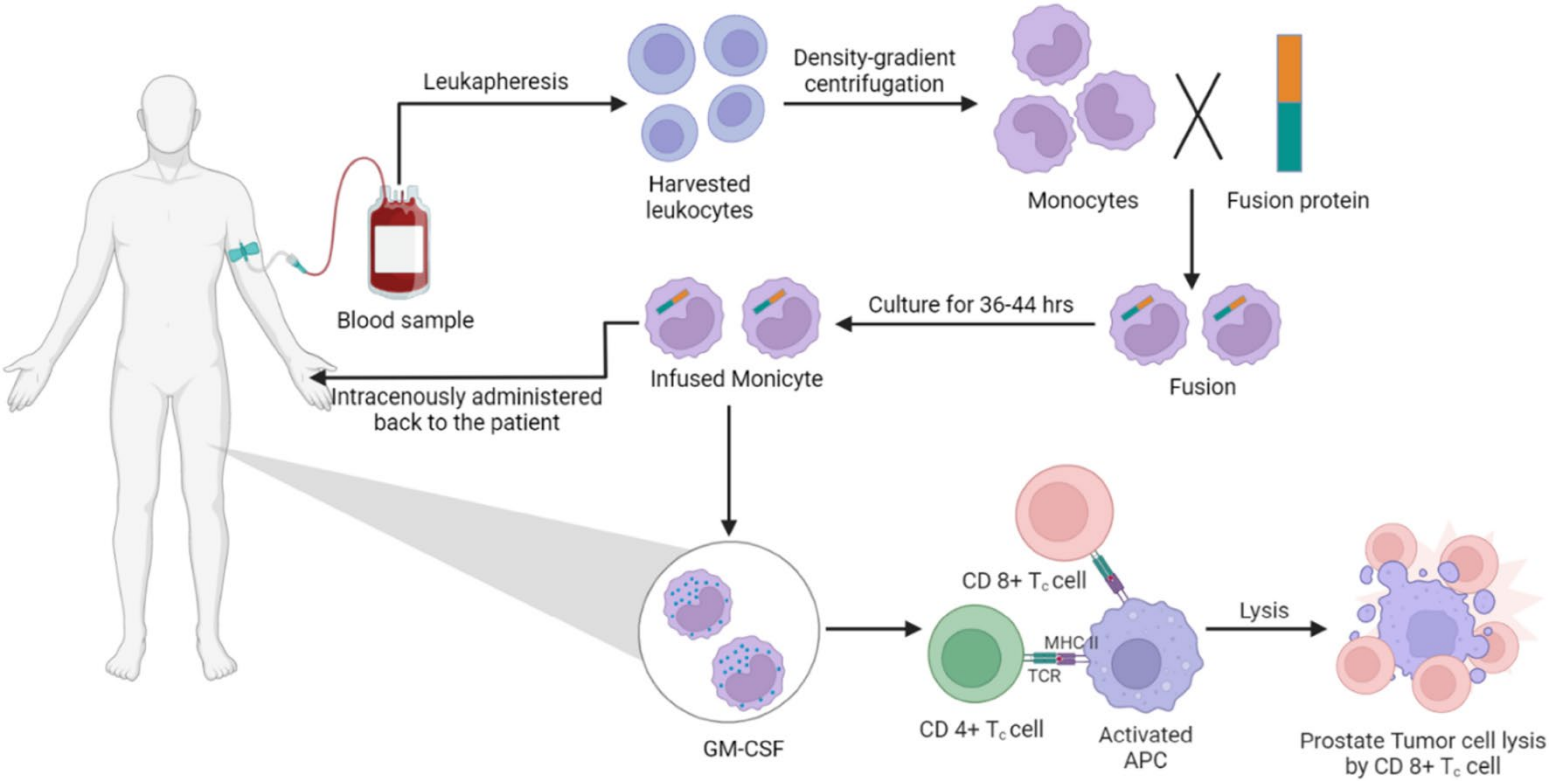
Mechanism of action of Sipuleucel-T. Sipuleucel-T is an autologous cellular immunological agent, here blood cells from prostate cancer patients are taken and processed through leukapheresis then density gradient centrifugation of leukocytes is done to get monocytes, monocytes are fused with fusion protein (PAP and GM-CSF) then it is culture for 36–44 h, infused monocyte is intravenously administered back to the patient. Infused monocyte having GM-CSF activates the APC that led to prostate tumor cell lysis by CD8 T cell. (GM-CSF-Granulocyte macrophage colony-stimulating factor, PAP-Prostatic acid phosphatase, APC- Antigen presenting cells)

Sipuleucel-T is a vaccine of autologous dendritic cells that unleashes an immune response against PAP antigen. This is an autologous active immunotherapeutic agent which has been reported to improve the survival of patients with mildly symptomatic PC [184]. It was the first US FDA approved therapeutic vaccine for cancer. It evokes patients’ immune systems to identify and combat cancer [185]. Detailed workflow of Sipuleucel-T immunotherapy is demonstrated in Fig. 4. The recommended dose for treatment is an intravenous infusion of three complete doses at an interval of two weeks. The most common after-effects include bleeding, bruising, pyrexia, fatigue, nausea, and headache [186]. This drug is not widely used because of its high production cost.

### G-VAX

G-VAX is a granulocyte–macrophage colony-stimulating factor (GM-CSF) gene-transfected tumor cell vaccine. It has been genetically modified for the expression of GM-CSF with the intention of increasing the differentiation and growth of dendritic cells [187]. This approach has the advantage of stimulate various TAAs without any pairing of HLA [188]. Though initial outcomes were promising, the successive results were negative against docetaxel from the phase III trials.

## Viral vector-based vaccines

Viral vector-based vaccines include the vectors obtained from oncolytic viruses. Such vectors can infect tumor cells and stimulate their death by antigen-presenting cells (APCs). Therefore, the APCs can produce TAAs which are responsible for the response of T cell [171]. A recombinant vaccine of Poxvirus which contains a PSA transgene with an HLA-A2 epitope, has been altered to enhance the immunogenicity of co-stimulatory molecules. The costimulatory molecules have been reported to be a B7-1 (CD80), an intercellular adhesion molecule 1 (ICAM-1 or CD54) and lymphocyte function-associated antigen-3 (LFA-3 or CD58) [189, 190]. The results published related to the PROSTVAC-VF have not shown a clear clinical benefit in treatment of PC patients [191].

## Immune checkpoint inhibitors

Tumor microenvironment (TME) is composed of various components which includes tumor cells, immune cells like myeloid-derived suppressor cells (MDSCs), tumor-associated macrophages (TAMs), tumor-associated neutrophils (TANs), tumor-associated dendritic cells (tDCs), and adoptive immune cells like the regulatory T cells (Tregs), extracellular matrix, stromal cells, vessels, soluble factors and physical properties [192]. However, the immunosuppressive microenvironment is composed of cellular and soluble components that promotes tumor progression and favors immune escape [192, 193]. Immune checkpoint inhibitors (ICIs) are referred as the monoclonal antibodies which are designed to target different receptors found in immune response [194, 195]. The most clinically proven ICIs are directed against PD-L1, PD-1, and CTLA4 [195]. PD-1 is a T cell transmembrane protein which has been reported to interact with its ligand (PD-L1) in the tumor cells [196]. PD-1 and PD-L1 are located on chromosome 9p24.1, and plays vital role in maintaining immune homeostasis [197], inside TME the activity of PD-1and PD L1 is seized by cancerous cells to escape immune surveillance [198]. Activation of T-cells are damaged due to interaction of overexpressed PD-L1 in cancer cell with PD-1 on tumor-infiltrating lymphocytes (TILs) which effects the TCR-signalling cascade by phosphorylating SHP-2 [199]. Transcriptional activation of PD-L1 is regulated by various transcription factors like MYC, STAT3, NF-κB, AP1, and HIF-1. Modification processes like ubiquitination, glycosylation, phosphorylation can affect the stability of PD-L1 protein in cancer cells so regulating the expression of PD-L1 protein [200]. High expression level of PD-1/PD-L1 has been reported in the PC cases, however, its role in response to ICIs remained debatable [201]. Inactivating the mutations in cyclin-dependent kinase-12 (CDK-12) have been reported to be directly related with an increased sensitivity towards immunotherapy. However, there are very few clinical data available to support the use of ICIs [202]. Only the presence of insufficient mismatch repair has been recognized by the clinical guidelines as a transversal agnostic for anti-PD1 therapy [203]. The synergistic effect of anti-D-1/PD-L1 and anti-CTLA-4 has been reported in renal cancer and melanoma [204, 205]. It has been shown that the expression of androgen receptor splice variant 7 (AR-V7) cause alterations in genes involved in DNA repair that made them more susceptible to ICIs [206]. In another study, the ipilimumab and nivolumab had synergistically effective in the cancer patients with the expression of AR-V7 [207]. CTLA-4 receptor has been reported to be present in the membrane of T lymphocytes. The stimulation of CTLA-4 receptor accelerates the inhibition of the function of T lymphocyte [208]. Ipilimumab has been shown to function as an anti-CTLA-4 agent and demonstrated a positive result for the treatment of PC in several studies [209]. The increase of myeloid-derived suppressor cells has been reported to be associated with the resistance to treatment [210]. The application of anti-PD-1/PD-L1 is limited to clinical trials in mCRPC [211]. Preclinical studies have shown that the over expression of PD-L1 were produced by the medication with Poly (ADP-ribose) polymerase (PARP) inhibitors [212]. Moreover, it has been reported that the sensitivity of NK cells can be enhanced by the treatment of Olaparib in PC [213]. In tumor microenvironment, the interaction between the immune response and angiogenesis pathway has been shown to favor the generation of an immunosuppressive state. The therapy with antiangiogenic agents has immunomodulatory effects which enable the response to ICIs [214].

## Gene therapy

Various strategies for gene therapy (GT) have been established through novel and advanced drug delivery systems. GT has shown significant potential for the cure of tumors in PC patients. Several types of GT are used to treat PC including suicide GT (SGT), tumor-suppressor GT (TSGT), anti-oncogene therapy (AOT), and immunomodulatory GT (IGT).

The concept of SGT is based on the killing of cancer cells by introducing a therapeutic gene in the cancer cell. After entry into the cancer cells, these SGTs have been reported to express and kill the cells without correcting the malignant mutations. It has also been shown that these SGTs have not affected the normal cells. SGT has been mainly divided into two major categories such as enzyme-based GT in cells. The enzyme-based SGT has been shown to suppress the proliferation of tumor [215]. In another study, Lee et al. (2020) have used the double SGT for the efficient treatment of PC using gemcitabine conjugated adenovirus [216].

TSGT has been achieved by introducing a wild-type gene into PC cells to suppress the proliferation of tumor [217]. The genes which have been usually studied for TSGT include p53, p21, and retinoblastoma [218, 219]. Successful GT has been achieved when all tumor cells remain transduced by the tumor-suppressor genes [220, 221]. The protein responsible for the tumor-suppression (p14ARF) has been used to regulate the activity of AR and modulate the level of p14ARF in prostate [218]. In another report, an Arv7-mediated CRPC has been created utilizing an active AR splice variant to inhibit the tumor [222]. Moreover, miR-21 has also been exploited to inhibit the proliferation of PC by targeting the tumor suppressor gene PTEN [223].

The capacity of immune system to detect and kill tumor cells is very low in humans. The immune system has been reported to be weakened due to the deficient expression of MHC antigens, which consequently lowers the T cells activation [224]. In this context, various immunomodulatory gene therapies have been established for the treatment of PC by using the gene vaccines [225]. Another type of immunomodulatory GT has been recognized as the intratumoral injection of cytokine genes using vectors [226].

Anti-oncogenes are made to target the specific tumor RNAs leading to the inhibition of tumor growth and proliferation. This therapeutic methodology has been used for the safe transgene delivery devoid of damaging normal cells and preventing the lysis of viral cells [227]. The cycle of cell lysis progresses until all the cancer cells are eradicated and make sure that the tumor is completely cured [228]. Adenovirus early region-1 is the most commonly used viral vector which has been reported to act as a good transgene carrier [229]. Due to the outstanding delivery of gene product, efforts has been made to conjugate with other therapeutic genes which exhibited strong promise for the treatment of PC [230].

## Nanotherapies

The use of nanotechnology has been expanded the modern scope for the treatment of diseases, and their diagnosis [231–233]. These nanocarriers have the potential to eradicate the cancer by targeted delivery of drugs and genes. There are several nanocarrier systems which have been used for enhancing drug delivery with higher biocompatibility of [234–236]. Major drug delivery systems in tumor treatment are polymeric spheres, liposomes, dendrimer, carbon nanotubes, mesoporous silica nanoparticles, virosomes, extracellular vesicles [237]. Apart from these therapeutic agents, the nanocarriers are being rapidly developed for the detection of tumor markers [238]. The aptamers have been reported to lack immunogenic toxicity and are easily synthesized [239, 240]. Self-assembled polymeric nanoparticles have been synthesized using the PLGA, and PEG and functionalized with Wy5a aptamer for the suppression of PC aggression [241]. Furthermore, these nanostructures loaded with doxorubicin has substantially eradicated the PC and delayed the growth of tumor in xenograft model [242]. The modification of nanoparticles with aptamers have been shown to increase the internalization into the PC cells [243]. In addition, hyaluronic acid-modified nanoparticles carrying epigallocatechin-3-gallate have been reported to significantly decrease the rate of proliferation of PC [244].

Ribonucleic acid interference (RNAi) has been utilized for silencing of the target gene. The application of nanocarriers for targeted delivery of RNAi has been recommended for enhancing the internalization of RNAi [245–247]. In another study, the siRNA-loaded with gold nanoparticles has been reported to penetrate the PSMA-over expressed PC cells [248]. The mesoporous silica nanoparticles has also been used to improve the gene silencing potential of siRNA in the tumor cells [249]. PLGA based nanocarriers have been made for androgen receptor-shRNA delivery for the suppression of PC [250]. The tumor cells has been reported to show increased sensitivity towards cisplatin by down-regulating the Lcn2 gene [251]. Sorrentino et al. have reported that interleukim-30 deletion using CRISPR/Cas9 reduces PC growth and elongates progression-free survival via upregulating SOCS3 and inhibiting the expression of IGF1 and CXCL5 [252]. Various nanocarriers have also been established for the delivery of anthracyclines in PC cells [253]. In addition, the polypeptide based nanocarriers loaded with doxorubicin have been shown to raise the oxidative damage, which eventually inhibited the metastasis of PC in mouse [254]. Several nanocarriers have been made to deliver the platinum-based agents to PC cells [255–257].

## Clinical studies

Clinical symptoms observed in PC may differ according to the level of the cancer. In other words, it depends on whether the lobe of prostate gland is affected and metastasized to other portions of body or not. In the case of locally advanced PC, cancer cells break out of the prostate gland, affecting nearby organs. Simultaneously, metastatic PC occurs when cancer metastasizes to the bones and lymph nodes. Patients with early PC are generally asymptomatic. Localized PC shows lower urinary tract symptoms (LUTS) in benign prostatic hyperplasia. Clinical manifestations of locally advanced PC include erectile dysfunction, painful ejaculation, sexual dysfunction [31, 32], hematuria, haematospermia, fatigue, low appetite, weight loss, nausea, vomiting, chronic bone pain in the pelvis, vertebrae, ribs, and hips. PSA testing and DRE enable the diagnosis of PC at early stages [258]. AR signaling have been reported to play a crucial role in initiation and the progression of PC [259]. Generally, localized PC is controlled by radical prostatectomy or radiation therapy with or without ADT [260]. In recent decades, substantial development has been made in the treatment of CRPC, such as abiraterone, apalutamide, enzalutamide, and darolutamide [261–265]. The new clinically approved agents by US FDA for diagnosis and treatment of PC are summarized in Table 3.

**Table 3:** US FDA approved therapeutic agents for the clinical use in the treatment of PC

S. No	Therapeutic agents used	Type of therapeutic	Date of US FDA approval	Date of EMA approval	Date of NMPA approval
1	^68^Ga-PSMA-11	Diagnostic radiopharmaceutical agent	Mar 2022		
2	^177^Lu-PSMA-617	Therapeutic radiopharmaceutical agent	Mar 2022		
3	Abiraterone	Endocrine therapeutic agent	Apr 2011	Sept 2011	Dec 2019
4	Cabazitaxel	Antineoplastic agents	Jun 2010	Mar 2011	
5	Dostarlimab-gxly	Immunotherapeutic agent	Aug 2021		
6	Degarelix	Endocrine therapeutic agents	Dec 2008	Feb 2009	July 2019
7	Denosumab	Bone-targeting therapeutic agent	Nov 2010	July 2011	May 2019
8	Darolutamide	Endocrine therapeutic agent	Jul 2019	Mar 2020	Feb 2021
9	Enzalutamide	Endocrine therapeutic agent	Aug 2012	Jun 2013	Nov 2019
10	Fluciclovine (18F)	Diagnostic radiopharmaceutical agent	May 2016	May 2017	
11	Olaparib	PARPi	May 2020	Nov 2020	Jun 2021
12	Padeliporfin	Antineoplastic agents		Sept 2017	
13	Pembrolizumab	Immunotherapeutic agent	May 2017		
14	Piflufolastat F 18	Diagnostic radiopharmaceutical agent	May 2021		
15	Radium-223 dichloride	Therapeutic radiopharmaceutical agent	May 2013	Nov 2013	Aug 2020
16	Relugolix	Endocrine therapeutic agent	Dec 2020	Mar 2022	
17	Rucaparib	Antineoplastic agents	May 2020		
18	Sipuleucel-T	Immunotherapeutic agent	Apr 2010	Sept 2013	
19	Zoledronic acid	Bone-targeting therapeutic agent	Feb 2002	Mar 2001	Dec 2018

Metastasis to bone has been successfully treated using the bisphosphonates, radium 223, and receptor activator of NFκ-B ligand inhibitor denosumab [121, 148, 266–269]. Several PARPi (rucaparib, olaparib, and talazoparib) has been evaluated in clinical trials for mCRPC [270–273]. PARP is responsible for repair of DNA damage [274]. Moreover, the early clinical studies focused to target ICIs, such as CTLA4, PD1 or PD-L1 have been evaluated [207, 275–278]. The PSMAs are greatly expressed in the cell membranes of PC [279]. Thus, PSMA targeting small molecules have been evaluated for their impact on PC cells in several clinical investigations [280–290]. Single-agent medication with the PI3K/AKT/mTOR inhibitors or in combination with inhibitors of AR signaling have also been studied in several studies [291–297]. The therapeutic agents targeting other signaling pathways, such as wingless-type protein signaling, CDK, p53, vascular endothelial growth factor, endothelin A receptor, receptor tyrosine kinases, epidermal growth factor receptor, fibroblast growth factor receptor, proto-oncogene tyrosine-protein kinase Src, transforming growth factor beta, and mitogen-activated protein kinase, have also entered clinical trials [298–309]. In recent years the several advancements have been done for the treatment of the PC. Despite these advances, current options for the treatment of PC have many limitations. Therefore, more specific treatment and targeted approaches are required for novel a better therapeutic possibility.

## Recommendations and guidelines

According to the European Association of Urology (EUA) 2020 guidelines, men with a PSA level of < 10 ng/mL are considered low-risk, PSA 10–20 ng/mL as intermediate-risk, and PSA > 20 ng/mL as high-risk [310]. The European Society of Medical Oncology recommends that risk calculation and mp-MRI be performed before a biopsy is performed. They also suggested performing transperineal biopsies instead of transrectal biopsies [311]. According to the EUA, there is not currently enough data to promote the use of ConfirmMDx for rebiopsy. Thus, due to the lack of evidence regarding the clinical utility of ConfirmMDx, its routine use is not recommended [312].

## Conclusions and future perspectives

PC has become a global burden because of the increasing number of patients and deaths. It is the second most commonly diagnosed cancer worldwide. Despite such a high prevalence of PC, its mortality rate is less because of PSA screening. In addition to recent screening techniques like DRE, ultrasound, and mp-MRI, liquid biopsy is an emerging diagnostic tool. The correct combination of chemotherapy drugs, such as docetaxel cabazitaxel, with ADT drugs, such as enzalutamide abiraterone, has improved OS in patients with mCRPC. Advancements in treatment strategies by analyzing cancer prognosis and patient preferences have helped lower mortality and increase the QoL of PC survivors. There appear to be a number of future therapies, such as the DNA repair pathway, platinum-based chemotherapy, and PARP inhibition. Further studies and phase trials are needed to develop therapies with fewer side effects. The use of radiotherapy and prostatectomy during an early stage of PC affects QoL of survivors. Therefore, improved therapeutic technologies will help to minimize the side effects of treatment.

## Data Availability

The material supporting the conclusion of this review has been included within the article.
